# Appointment of new section editors

**DOI:** 10.1002/1878-0261.12586

**Published:** 2019-10-31

**Authors:** Julio E. Celis

In the year following the introduction of our first section editors, the number of submissions to *Molecular Oncology* has continued to grow apace. To better reflect the increasing size of the journal, I am now pleased to announce the appointment of two new section editors, Heike Allgayer and Joachim Schüz. Heike is Full Professor and Director, Department of Experimental Surgery‐Cancer Metastasis and Vice Director, Centre for Biomedicine and Medical Technology Mannheim (CBTM), Medical Faculty Mannheim, Ruprecht Karls University, Heidelberg, Germany. Heike’s main research areas of expertise include metastasis, translational research, and targeted therapy. Joachim Schüz is Head of Section (Environment and Radiation) at the International Agency for Research on Cancer (IARC), Lyon, France, and is currently coordinator of Cancer Prevention Europe (CPE), a consortium of leading organizations across the whole of Europe that aims at reducing morbidity and mortality from cancer in European populations through prevention and earlier diagnosis of the disease. Joachim’s areas of expertise include cancer epidemiology, radiation epidemiology, environmental exposures, and biostatistics.

We are delighted to welcome both editors to the team, as their experience and dedication will be instrumental to the continued success of the journal.

This month also sees the appointment of Maria Papatriantafyllou as a full‐time Editorial Manager of *Molecular Oncology*. Maria will manage the day‐to‐day operation of the journal and will work closely with our authors, reviewers, editors, and publishing partners. She brings considerable editorial experience to our journal, having worked previously on *Nature Reviews Immunology* and *Nature Reviews Molecular Cell Biology*, as well as our sister journal, *FEBS Letters*. Maria obtained her PhD at the German Cancer Research Centre, where she studied the regulation of T‐cell responses by Dickkopf‐3.

I look forward to working with Maria and would like to warmly welcome her to the journal on behalf of the whole team.

Finally, our sister journal, FEBS Open Bio, has also seen a rise in submissions. Duncan Wright, who has previously worked as Editorial Associate across both journals, will be leaving *Molecular Oncology* to take up a full‐time position as Editorial Manager of *FEBS Open Bio*. It has been a great pleasure for me to work with such a talented young colleague.

## Section editors

Heike Allgayer:

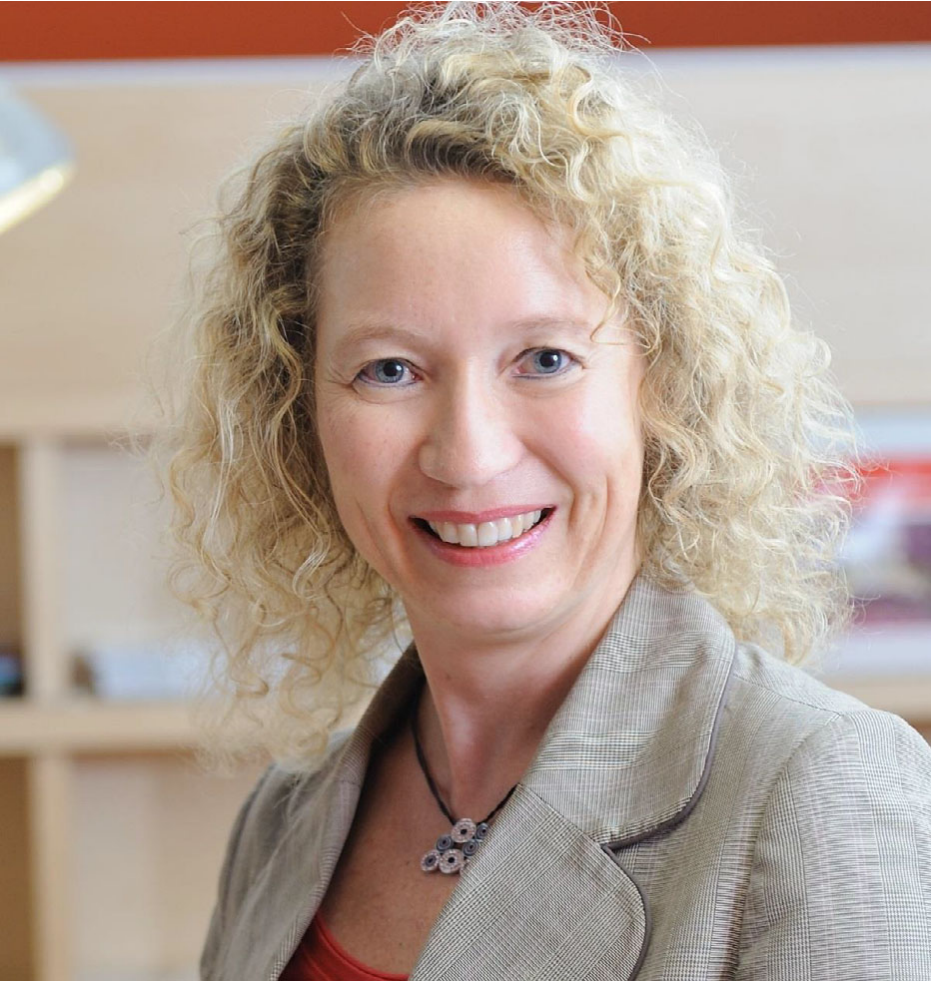

Heike Allgayer received her MD and Dr. med. (summa cum laude) at Ludwig‐Maximilians University Munich in 1995/1996, and her PhD (in molecular biology) at the University of Houston‐MD Anderson Cancer Center in 1999. As a board‐certified surgeon and molecular biologist, she sees her mission in combating cancer metastasis, bridging basic molecular research with clinically relevant hypotheses as a dedicated translational researcher. Her primary research interests include molecular determinants defining metastasis, tumor‐associated proteases, microRNAs in metastasis, molecular determinants promoting personalized therapy against metastasis, molecular staging, translational metastasis research, and metastasis prevention.

Her publications and research activities (her most cited original article currently showing about 3000 citations) have received more than 20 scientific awards, including the Research Award of the Alfried‐Krupp‐von‐Bohlen‐und‐Halbach‐Foundation, the Langenbeck and Sauerbruch Research Award, the Young Investigator Award of the American Association of Cancer Research (AACR), the Research Award Lecture of the European Association for Cancer Research (EACR), the Johann‐Georg‐Zimmermann Award for Cancer Research, and the Research Award of the Ingrid zu Solms‐Foundation, for which she currently also serves as the Chair of the Fellowship of the Awardees. She is active member of, for example, the AACR (served as member of the AACR Surgical Oncology Task Force and the Program Committee for the AACR Annual Meeting 2017), ASCO, and EACR (invited member of the EACR Ambassadors and EACR Conference Program Committee), member of the EORTC Advisory Board for Translational Research, Academia.Net, founder of the International Meeting series on ‘Molecular Staging of Cancer’, and Associate Editor of the International Journal of Cancer, amongst other appointments. In 2017, she became elected as a Fellow of the European Academy of Cancer Sciences (EACS).

Joachim Schüz:

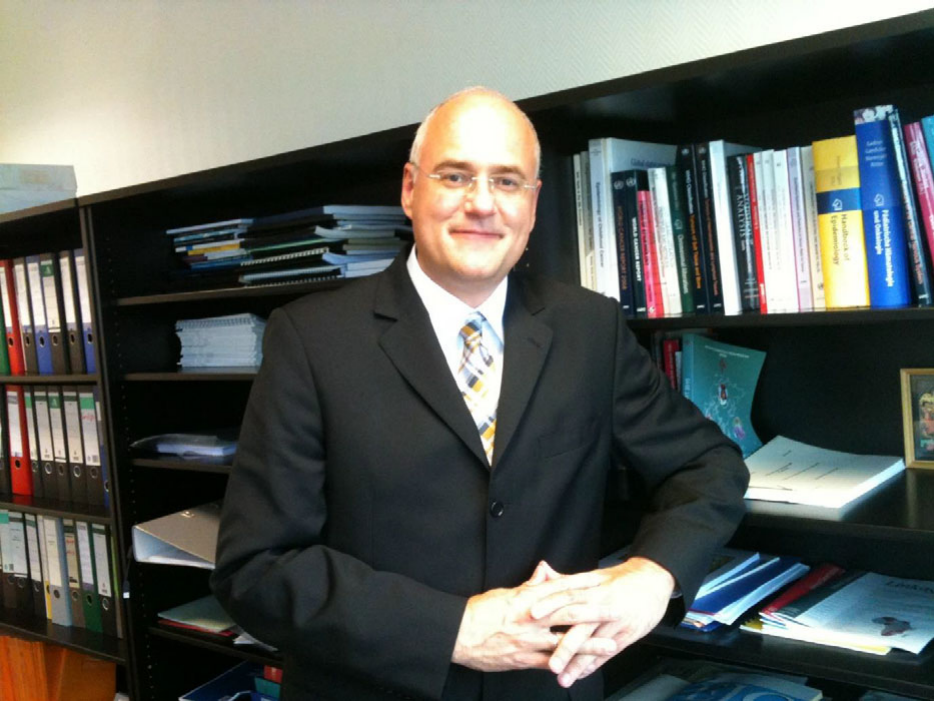

Joachim Schüz completed his PhD in Epidemiology in 1997 and his professorial thesis in 2002 (‘Habilitation’ in Germany) on the topic of ‘Epidemiology of childhood leukemia’. He worked in cancer epidemiology throughout his career, first with a focus on childhood cancer and radiation‐related cancer (including non‐ionizing radiation), but later broadening to environmental, occupational, and radiation‐related cancers. Other notable positions and functions were: a member of the Scientific Committee on Emerging and Newly Identified Health Risks (SCENIHR) of the European Commission, President of the Bioelectromagnetics Society, and Honorary Professor at the London School of Hygiene and Tropical Medicine. Through other committees and advisory boards in addition to the IARC mandate of consulting governments in cancer control, he has several links with cancer prevention and how to implement what we know about the causes of cancer in effective cancer prevention. The most noteworthy activities in this context are Co‐Principal Investigator of the 4th edition of the European Code against Cancer and, since 2018, being chairman of the recently created Cancer Prevention Europe consortium.

